# Implementing Cultural Safety in Research Methodology: The Co‐Design Process of a Brief Therapeutic Intervention for Aboriginal and Torres Strait Islander Young People Who Engage in Self‐Harm and/or Suicidal Behaviours

**DOI:** 10.1111/ajr.70152

**Published:** 2026-02-06

**Authors:** Craig D'Mello, Helen Milroy, Alana Papageorgiou, Mathew Coleman, Patricia Dudgeon, Paulette Anderson, David Batty, Ashleigh Lin

**Affiliations:** ^1^ School of Population and Global Health The University of Western Australia Perth Australia; ^2^ The Kids Research Institute Nedlands Australia; ^3^ School of Psychiatry The University of Western Australia Perth Australia; ^4^ Rural Clinical School of WA University of Western Australia Perth Australia; ^5^ Australian Research Centre in Sex, Health, and Society La Trobe University Bundoora Australia; ^6^ West Australian Country Health Service Perth Australia; ^7^ School of Indigenous Studies The University of Western Australia Perth Australia; ^8^ Youth Focus Meekatharra Australia; ^9^ Geraldton Regional Aboriginal Medical Health Service Geraldton Australia

**Keywords:** Aboriginal and Torres Strait Islander, cultural safety, Indigenous, self‐harm, suicide, young people

## Abstract

**Background:**

Aboriginal and Torres Strait Islander Peoples share rich cultural traditions unrivalled across the world; however, the continued impact of colonisation led to sustained, profound trauma that has spanned generations. With Aboriginal and Torres Strait Islander people presenting to hospital emergency departments (ED) for self‐harm and suicidal behaviours at a rate 2.9 times higher than non‐Indigenous people, there is a need to develop culturally appropriate interventions to address this growing problem.

**Objective:**

This paper sought to describe the co‐design process of culturally adapting a brief therapeutic intervention for Aboriginal and Torres Strait Islander young people who display self‐harm and/or suicidal behaviours. The adaptation focus was Therapeutic Assessment (TA), a brief intervention provided to young people who present to the ED with self‐harm.

**Setting:**

The process was split into two phases located in Geraldton and Meekatharra, two communities in the Mid‐west of Western Australia.

**Participants:**

In phase one, three male Aboriginal young people (aged between 16 and 19 years old) and eight Aboriginal Elders participated in two yarning circles run on one day. In phase two, 26 Aboriginal young people (aged between 12 and 25 years old), five Aboriginal senior members of the community and one Aboriginal carer participated in a combination of small yarning circles and/or single interviews.

**Results:**

This paper describes the elements of the culturally safe process of adapting a brief intervention for Aboriginal and Torres Strait Islander young people experiencing self‐harm and/or suicidal behaviours.

**Conclusion:**

Two points are important to note. The first is that implementing a culturally safe process can be an outcome in itself, and second, that the principles supporting cultural safety can assist in evaluating how non‐Indigenous researchers implement this process.

## Key Points

**What Is Already Known About This Topic:**

- Aboriginal and Torres Strait Islander young people are over five times as likely to die by suicide than their non‐Indigenous peers.
- Aboriginal and Torres Strait Islander young peoples living in rural communities define suicidal behaviours much more broadly than in traditional Western understandings.
- A culturally safe outcome can only be derived from a culturally safe process and adherence to fixed conventions are not helpful when decolonising research methodologies.

**What This Topic Adds:**

- By employing a culturally safe approach (e.g., taking time to explain the key terms and the interview process), participants appeared to demonstrate a decrease in heightened emotional affect as interviews and yarning cirlces progressed.
- It was observed that through employing a culturally safe co‐design approach, participants voluntarily disclosed increasingly more sensitive information as interviews and/or yarning circles progressed.
- By remaining flexible and providing greater options (e.g., having the interview on Country or in an office, option of bringing a support person, sharing in a group or individually), it positioned participants with greater autonomy and control which elicited richer data.

## 1 Introduction

Aboriginal and Torres Strait Islander Peoples share rich cultural traditions unrivalled across the world for over 60 000 years. Before British colonialism, there were between 300 000 and 950 000 Aboriginal people living in Australia, each living securely within a relationship to the physical, spiritual and social environments [1, 2]. Specifically, connection to Country is deep and spiritual, crucial to identity as Aboriginal and Torres Strait Islander Peoples. The health and wellbeing of Aboriginal and Torres Strait Islander people has been affected by the continued impact of colonisation and the subsequent genocide and forced removal of children from their families. This has led to sustained, profound trauma spanning generations [1, 3]. Being robbed of self‐autonomy has resulted in feelings of powerlessness and limited self‐efficacy, both of which are linked to suicidal behaviours [4, 5, 6].

Suicide is a complex, global phenomenon that results in 800 000 lives lost worldwide each year, with self‐harm presentations and recent suicidal behaviour the strongest predictors of death by suicide in young people [7, 8, 9]. Aboriginal and Torres Strait Islander young people aged between 15 and 24 years old are over five times more likely to die by suicide than their non‐Indigenous counterparts; that figure almost doubling for those aged 10–14 years old, who are almost nine times more likely to die by suicide than non‐Indigenous children [10, 11].

Aboriginal and Torres Strait Islander young people living in rural communities define suicidal behaviours much more broadly than in traditional Western understandings. In a recent community‐based study, Aboriginal and Torres Strait Islander people defined suicidal behaviour to include ‘reckless and/or self‐destructive behaviour’ and ‘hanging around certain places within the community’ [12, 13]. In a recent study investigating all suicide deaths of people aged 10–19 years in Queensland (1st January 2001–21 December 2015), Aboriginal and Torres Strait Islander young people were almost twice as likely to die by suicide if they lived in regional and remote areas (24 per 100 000 persons) compared to major cities (14 per 100 000 persons) [14].

Taken together, this raises two important points when investigating suicidal behaviours in regional Aboriginal and Torres Strait Islander communities. First, that traditional Western definitions of suicidal behaviours may not align with those held by some Aboriginal and Torres Strait Islander communities, and second, that there appears to be community and contextual factors specific to those experienced by Aboriginal and Torres Strait Islander people living in these communities. With Aboriginal and Torres Strait Islander people presenting to the Emergency Department (ED) for self‐harm and suicidal behaviours at a rate 2.9 times higher than non‐Indigenous people, there is a distinct need to develop a culturally appropriate therapeutic intervention to address this growing problem [8, 15].

This paper describes the co‐design process of culturally adapting a brief therapeutic intervention for Aboriginal and Torres Strait Islander young people who display self‐harm and/or suicidal behaviours. The adaptation focus was Therapeutic Assessment (TA), a brief intervention provided to young people who present to the ED with self‐harm. TA has been associated with a significant increase in treatment engagement of young people who present with self‐harm [16, 17]. Here we describe what was done to implement cultural safety as part of the adaptation process and how this culturally safe process was implemented.

### 1.1 Prior Adaptations in Suicide Prevention for Aboriginal and Torres Strait Islander People

While culturally adapting a mental health assessment is an important outcome, the implementation of a culturally safe co‐design process is just as important [1, 18]. Cultural safety is a dynamic construct; it is the individual and institutional knowledge, skills, attitudes and competencies needed to deliver best practice healthcare for Aboriginal and Torres Strait Islander individuals, families and communities [19, 20]. While cultural safety is ultimately determined by the person and their family, depending on the community and context, it requires clinicians and researchers to engage in collaborative communication. This is built on genuine active listening as the foundation for building trust and subsequently advocating for the Aboriginal and Torres Strait Islander person's autonomy [20, 21]. Two important points must be noted. One, a culturally safe outcome can only be derived from a culturally safe process, and second, adherence to fixed conventions is not helpful when using decolonising research methodologies [1].

Previous studies have sought to implement a culturally safe co‐design process when culturally adapting specific training programs and interventions in the field of suicide prevention. An example is the Indigenous Network Suicide Intervention Skills Training (INSIST) program, a suicide intervention training program targeting at‐risk Indigenous youth [22]. INSIST utilised culturally safe elements in the design, implementation and empirical evaluation of the program. The strengths of this cultural adaptation process include the incorporation of ‘yarning’ (a traditional Aboriginal and Torres Strait Islander conversational process that involves informal, relaxed sharing of narratives as a method of acquiring and developing a knowledge base [23, 24]) using a semi‐structured format through a series of questions to facilitate discussion and having multiple workshops.

While the implementation of a yarning methodology can establish cultural safety within the cultural adaptation process, wide consultation and the implementation of varied communication methods is also crucial. The Stay Strong Plan, developed by the Aboriginal and Islander Mental health initiative (AiMhi), is a culturally adapted low intensity cognitive behavioural therapy intervention for reducing psychological distress and depression in Aboriginal and Torres Strait Islander people which used culturally safe elements and a community‐based participatory design research [25, 26]. Implementation of an Aboriginal and Torres Strait Islander youth reference group, 16 co‐design workshops and a dynamic community consultation process (using mock‐ups, group discussions, visual flow diagrams and storyboards) were key strengths in the cultural adaptation process.

## 2 Methods

In this section, we identify seven methodological considerations crucial to the implementation of cultural safety as part of the co‐designed adaptation process.

1. Community consultation
2. Researchers' lived experiences
3. Indigenous governance (and training for non‐Indigenous researchers)
4. Co‐design processes and ongoing community engagement
5. Context.
6. Self‐reflexivity and feedback
7. Ethics

### 2.1 Community Consultation

Prior to implementing any adaptation, engagement and consultation with the community is fundamental. The initial step of engaging with multiple Mid‐west health service workers (i.e., Geraldton Regional Aboriginal Medical Service, Headspace, Youth Focus) and the creation of an Advisory Group comprised of eight Aboriginal Elders allowed us to ask three important questions: (1) does this cultural adaptation fit with the key priority areas of the community? (2) does this cultural adaptation fit the community needs? and (3) will this cultural adaptation lead to improvement within the community? While this was a crucial first step, the importance of consultation did not diminish as the adaptation process evolved. The Advisory Group participants were originally recruited to advise in the creation of the questions, but the rich data they provided was pertinent to other parts of the study and as such, they have been included as data sources. Informed consent was provided prior to the commencement of the Advisory Group to use their data in a participatory way.

### 2.2 Researchers' Positionality

This paper emerged as part of a PhD research project that aims to understand and support Aboriginal and Torres Strait Islander young people experiencing self‐harm and suicidal behaviours. The first author, lead researcher and PhD student C.D. is a non‐Indigenous young male of Anglo‐Indian heritage who lives on Wadjuk Noongar Country and works professionally supporting Aboriginal and Torres Strait Islander people in regional and remote areas of Western Australia. He is generally registered as a Psychologist and Social Worker and has previous experience facilitating Social and Emotional Wellbeing workshops with Aboriginal Elders, community members and stakeholders.

The other members of the research team included: H.M. is a Palyku woman of the Pilbara region, born and educated in the Wadjuk Noongar Country. A.P. is a Greek Australian, youth mental health researcher living and working on Wadjak Noongar Country and born and raised on Lutruwita. M.C. is of Anglo‐Australian heritage working as a clinical academic and psychiatrist on Southern Yamatji Country. P.D. is a Bardi woman from the Kimberley region. P.A. is of Anglo‐Australian heritage and a Registered Nurse, living and working on Yamatji Country in Meekatharra. A.L. was born in South Africa and raised on Wadjuk Noongar Country; she is a youth mental health researcher.

### 2.3 Indigenous Governance (Training for Non‐Indigenous Researchers)

In line with Aboriginal Participatory Action Research methodologies [27, 28] the cultural adaption to TA was co‐developed with Aboriginal investigators and involved partnership with a regional Aboriginal organisation (i.e., Geraldton Regional Aboriginal Medical Service). Indigenous governance was strengthened by two senior Aboriginal researchers who supervised the non‐Indigenous researcher to maintain cultural oversight. In Geraldton, the non‐Aboriginal lead researcher partnered with a local Aboriginal co‐researcher to collectively identify community risk and protective factors, build trust and rapport with community members, and create an Advisory Group comprised of eight Aboriginal Elders. The Advisory Group provided local Indigenous leadership to the cultural adaption while discussing their unique lived experience of supporting Aboriginal and Torres Strait Islander young people who experience self‐harm and/or suicidal behaviours. This fostered localised knowledge generation, key in culturally adapting TA for communities in the middle of Western Australia.

Learning how to implement cultural safety, as a non‐Aboriginal researcher, is a continuous process built on collaboration, curiosity and self‐reflexivity. While the non‐Aboriginal lead researcher had significant clinical experience supporting Aboriginal and Torres Strait Islander people, participation in a Cultural Exchange Program [29] supported further knowledge growth in implementing cultural safety. This particular Cultural Exchange Program was developed, led and run by Aboriginal people where non‐Indigenous researchers met with a group of Aboriginal Elders who shared stories, meals and feelings, with an aim on learning how to work with Aboriginal and Torres Strait Islander people in culturally safe ways. This way of learning highlighted the positive impact of social yarning, truth telling, deep listening and knowledge sharing.

### 2.4 Co‐Design Processes and Ongoing Community Engagement

The initial step involved engaging with Aboriginal Elders (four male and four female) with varied lived experience of suicidal thoughts, or caring for someone experiencing self‐harm and/or suicidal behaviours. This was conducted by the lead researcher and a co‐facilitator, an Aboriginal male with long‐standing roots in the Geraldton community with vast experience facilitating groups with varied interpersonal dynamics. The two facilitators prepared for the Advisory Group by discussing who would introduce which sections and the role of each facilitator. During the Advisory Group, the process of TA was discussed in addition to how non‐Indigenous researchers can implement TA as part of a culturally safe process, the aim being to develop the questions posed to those participating in subsequent yarning circles and interviews in the next phase of the project. As part of this process, the Advisory Group noted that the overall structure and model of TA aligned with their experience caring for Aboriginal young people with self‐harm and/or suicidal behaviours, and that major structural changes were not required to fit with community understanding of suicide and/or self‐harm. Minor changes (e.g., terminology, process, communicative aids) were required.

### 2.5 Context

The process of culturally adapting TA was split into two phases. The first phase focused on Geraldton, a large regional coastal town in Western Australia, with the second phase moving inland to Meekatharra, a small remote community located within the Mid‐West. Three male Aboriginal young people (aged between 16 and 19 years old) participated in a yarning circle in Geraldton. The yarning circle was co‐facilitated by the same researchers that facilitated the Advisory Group and lasted for 90 min.

Phase two was conducted in Meekatharra. Twenty‐six Aboriginal young people (aged between 12 and 25 years old) participated in a combination of small yarning circles and/or single interviews. In addition, five senior Aboriginal community members and one Aboriginal carer participated in single interviews or small interviews with two people. Of those six, four were female and two were male. Two Aboriginal senior members opted to provide a second interview with a third Aboriginal senior member providing three repeat interviews. Interviews were facilitated by the lead researcher, with each session lasting for approximately 40 min (ranging from 20 to 90 min).

For both phases, the process was flexible in different components and allowed for participants to feel in control of the process. This trauma‐informed way of working helped to facilitate cultural safety as part of the process. Participants were given the choice of environment (e.g., office setting, on Country), inclusion of a support person, they were provided opportunities for breaks, and there was explicit consent that they could withdraw at any time or refuse to answer any question. The facilitator explored the participants' personal lived experience of self‐harm and/or suicidal behaviours and held space for truth telling, knowledge sharing and grieving. Both interviews and yarning circles were audio recorded with participants' permission for later transcription.

### 2.6 Self‐Reflexivity and Feedback

One important technique to enhance the trustworthiness of the data was to present a summary of the discussions to participants [30]. In Geraldton, a two‐page summary sheet in plain English was provided to participants after the results obtained from the yarning circle were checked with participants for accuracy prior to the conclusion of the session. In Meekatharra, senior members of the community that participated were provided the opportunity to review the results obtained from the interviews and provide additional feedback. This provided an opportunity for participants to check the accuracy of the data through regular consultation. The method of triangulation was adopted to validate multi‐perspective data [31, 32]. The triangulation was also adopted via semi‐structured interviews with Aboriginal young people, yarning circles with Aboriginal Elders and the establishment of an Advisory Group. All three were employed so that robust, multi‐perspective data could be gathered and validated, and so these findings could be fed back to participants and subsequently ‘double‐checked’ by Aboriginal people. Adjustments were made to the data as part of this process including the refinement of key themes and the creation of sub‐themes whilst also allowing for comparisons to be made between groups (e.g., Elders and young people). While the data was not modified, the different perspectives shared allowed for a richer interpretation of the data.

This provision of bilateral feedback resulted in Aboriginal participants having an increased inclusion in the research process whilst prioritising Aboriginal knowledge. This adheres to the standards for conducting ethical research with Aboriginal and Torres Strait Islander people outlined in the Australian Institute of Aboriginal and Torres Strait Islander Studies (AIATSIS) Code of Ethics [33], the Aboriginal Health and Medical Research Council (AHMRC) Key Principles [34] and the National Health and Medical Research Council (NHMRC) Values and Ethics Guidelines [35]. This feedback process was continual and occurred at various points during the process (e.g., asking participants for feedback post interview, feeding back knowledge and ideas shared through interviews/yarning circles back to participants, allowing opportunities for multiple repeat interviews).

### 2.7 Ethics

This project received ethics approval from the Western Australian Aboriginal Health Ethics Committee (HREC 1142), with the institution (The University of Western Australia) recognising this existing approval. Informed consent was obtained prior to the commencement of each interview/yarning circle. Confidentiality and the limits to confidentiality when working in a group and individual setting were discussed. Participants were notified that all data would be kept on password protected computers after being removed from recording devices used for transcription. One participant did not give consent for their interview to be recorded; comprehensive session notes were taken in this instance.

## 3 Results

Results of this paper will be presented by describing how the process of implementing cultural safety within the study was an outcome in itself. Specifically, we will describe the process of engaging with Aboriginal Elders, young people and their carers to co‐design a culturally adapted brief intervention for Aboriginal young people who experience self‐harm and suicidal behaviours and how this process was a culturally safe outcome. As such, findings are structured loosely on the principles supporting culturally safe evaluation [36]. Further, the process of implementing cultural safety within the study's methodology, described below, is based on previously implemented culturally safe methodologies when working with Aboriginal and Torres Strait Islander people (Yarning, Dadirri and Ganma) [28, 37].

### 3.1 Benefits and Community Priorities

In line with the principles supporting culturally safe evaluation, preliminary discussions were had with multiple people regarding the anticipated benefits of the cultural adaptation to Aboriginal and Torres Strait Islander people. Rather than engage in this discussion only with the Advisory Group, stakeholders from community and Indigenous health organisations were also consulted (e.g., Geraldton Regional Aboriginal Medical Service, Youth Focus, Headspace) on whether this cultural adaptation would lead to benefit within the community and align with their priorities. The lead researcher consulted with multiple stakeholders and Elders from the community in a collaborative manner. This was done by first remaining curious to the multiple shared and unique experiences of others by employing open‐ended questions with a focus on the community's priorities rather than the researcher's priorities. Consequentially, the non‐Indigenous lead researcher appreciated their limited capacity to attend to all community priorities and subsequent co‐design based on collaboration was key to result in a beneficial outcome.

### 3.2 Indigenous Governance and Co‐Design

Given the context of decades of exploitative research of Aboriginal and Torres Strait Islander Peoples, the anticipated barriers likely to arise when non‐Indigenous researchers engage with Aboriginal and Torres Strait Islander young people was discussed with the Advisory Group in Geraldton and senior Aboriginal community members in Meekatharra. These initial conversations resulted in the non‐Indigenous researcher gaining a deeper appreciation for Aboriginal and Torres Strait Islander peoples' right for self‐autonomy. Arguably, a culturally safe co‐design process involved the non‐Indigenous lead researcher accepting (1) the context in which their research exists, (2) the past (and often traumatic) experiences Aboriginal and Torres Strait Islander people have had engaging with non‐Indigenous researchers, and (3) the limitations that the non‐Indigenous researcher may have with regards to co‐design experience [18]. This approach appeared to evidently strengthen the partnership between the non‐Indigneous lead researcher and the Aboriginal individuals and organisations that governed and participated in the project.

By remaining cognisant of these key understandings, the non‐Indigenous researcher was better able to remain open and understanding, which allowed for a more genuine partnership to be built. While the non‐Indigenous researcher had significant past experiences supporting Aboriginal and Torres Strait Islander people in a clinical context, the limitations in their experience was explicitly noted to participants as part of the interview process. This approach allowed for greater transparency and honesty from the very commencement of the research process and solidified the importance of including an Aboriginal Community Researcher to co‐facilitate the Geraldton yarning circle. Validating the past negative experiences many Aboriginal participants shared with non‐Indigenous researchers created an opportunity to provide participants with a corrective, therapeutic experience. Tactful use of silence and adapting specific non‐verbal communication styles (e.g., softening of facial expressions/vocal tone, open and engaging body language) to suit context were essential. It was observed that through employing a culturally safe co‐design approach participants voluntarily disclosed increasingly more sensitive information, with some participants going even further by validating the emotions of the other participants in the yarning circle.

### 3.3 Truth Telling

As the research process progressed through to the Advisory Group, yarning circle and clinical interviews, the lead researcher solidified their understanding of the importance and process of deep listening. Dadirri, an Aboriginal word from the Ngangikurungkurr Peoples from Daly River in the Northern Territory of Australia, is a culturally informed concept that refers to the contemplative process of listening deeply within reciprocal relationships with the intent of bringing profound awareness [28, 38]. Dadirri is increasingly used as a culturally safe research methodology with Aboriginal people; with first‐hand accounts noting the healing practice as bringing peace, empowerment and ownership [38]. Initially, co‐facilitation of the Advisory Group and yarning circles involved the lead researcher taking a purposeful step back by remaining silent and allowing for participants to share their voices. As the process of listening deeply allowed for more truth telling, it was essential to (1) allow space for participants to process these truths and (2) to validate the shared experiences of participants who care for young people who experience self‐harm and/or suicidal behaviours. It became evident that showing participants you are listening deeply to their stories by modifying non‐verbal communication (e.g., subtle head tilts/nods and a softening of gaze in response to truth telling) allowed for harder truths to be told as this style of engagement appeared to validate the often‐distressing experiences that were shared.

### 3.4 Diversity

Co‐facilitation of Advisory Groups and yarning circles bring unique challenges that interviews do not. Arguably, it is important to not only listen deeply to participants during truth telling, but also create space and opportunity for quieter participants to talk where there is likely to be varied levels of engagement. Through the process of Dadirri, participants appeared to feel safe with the environment, as demonstrated through increased engagement and harder truths being told. This safety, and through therapeutically validating the shared and unique participant experiences, strengthened the connection between the non‐Indigenous researcher and Aboriginal participants. This connection was used to test the capacity for the non‐Indigenous researcher to take on a more active role by respectfully navigating conversation, creating space for quieter participants to speak.

Ganma, an Aboriginal word from the Yolgnu Peoples of Arnhem Land in the Northern Territory, refers to the respectful two‐way sharing of cultural knowledge between Aboriginal and non‐Aboriginal people [28]. Ganma involves each person remaining mindful of the others' individual and combined experiences and provides a pathway for connection and collaboration; essential to create new shared knowledge [28, 39]. Ganma, closely linked to Dadirri, is built on patience, deep listening and respect for each other's culture [39]. A two‐pronged approach was required to facilitate Ganma with the intent of allowing for more diverse truth telling. First, it was important to validate the emotions after a participant shared their truth. This then allowed for the non‐Indigenous researcher to gently guide conversation to less active members of the group without it appearing rude. This was achieved by validating the experience of one participant while slowly shifting eye contact to less active participants and asking if anyone had a similar experience. The combination of verbal and non‐verbal communication strategies successfully allowed for multiple, diverse perspectives and shared knowledge.

### 3.5 Time and Trust

An inherit outcome of Yarning and Dadirri is the increased time spent developing professional therapeutic relationships and building trust with each participant. If validating the experiences of participants is crucial to truth telling, then rushing the process would have been counter to this goal. Over 24 months were spent on engagement and co‐design, punctuated by lengthy stays within the Geraldton and Meekatharra communities to build a genuine connection and an understanding of local barriers, priorities and concerns. Within yarning circles, adequate time was spent on social yarning and developing a genuine connection with each participant. This was demonstrated by asking open‐ended questions and taking time getting to know and connect with each participant through a common and shared interest [23, 40]. The lead researcher also spent significant time with each participant prior to the commencement of the interview, describing what the interview would focus on and explaining the Participant Information and Consent Form. This decolonising methodological approach created a genuine connection between the lead researcher and Aboriginal participants which helped to build trust and safety, and consequently allowed for greater sharing of knowledge and truth telling. It was evident in participant discussions that by utilising this culturally safe approach (e.g., taking time to explain the key terms and the interview process), participants appeared to demonstrate a decrease in heightened emotional affect as the interview progressed, as demonstrated through a relaxation in body language.

### 3.6 Adaptability and Flexibility

Implementing a culturally safe co‐design process rests on gathering data flexibly by providing multiple ways for participants to share sensitive personal information. The non‐Indigenous researcher demonstrated flexibility in process by allowing the participants multiple different options (e.g., having the interview on Country or in an office, providing the option of bringing a support person, sharing in a group or individually). Remaining flexible and providing greater options positioned the participant with greater autonomy and control. This trauma‐informed way of working appeared to create a safer space within the yarning circles/interview which allowed for greater Ganma.

Several challenges arose during the co‐design process that required flexible, dynamic responses and reflection in‐action. One challenge was the suspected presence of undiagnosed neurodevelopmental disorders (i.e., Attention Deficit Hperactivity Disorder, Fetal Alcohol Spectrum Disorder, Autism Spectrum Disorder) for some of the young people interviewed. Given the nuances of therapeutically supporting (and gathering retrospective information from) people with neurodevelopmental disorders, it became apparent that adhering to fixed conventions and protocols would not be helpful. Some participants required longer time to process and appraise information; others required a small break during the interview. Many found it difficult to verbally express their feelings, thoughts and behaviours as they experienced them in that moment. The implementation of a culturally safe research process encouraged this shared decision‐making in session (e.g., the option of a break), adaptability (e.g., provision of multiple shorter sessions in lieu of one big session) and respect (e.g., purposeful silences to hold space for non‐verbal expressions of emotion). It was evident in participant discussions, and by closely monitoring the participant's affect throughout the interview, that employing a flexible approach fostered greater cultural safety within the process.

## 4 Discussion

This paper describes the elements of the culturally safe process of adapting a brief intervention for Aboriginal and Torres Strait Islander young people experiencing self‐harm and/or suicidal behaviours. This specific methodology was based on previous Indigenous methodologies (i.e., Yarning, Dadirri, Ganma) [28, 37], with the findings loosely structured on several principles supporting culturally safe evaluation (i.e., time, decision‐making, diversity, respect, adaptability, benefits) [36]. Two points are important to note. The first is that implementing a culturally safe process can be an outcome in itself, and second, that the principles supporting cultural safety can assist in evaluating this process.

Viewing a culturally safe process as an outcome is not novel. There have been several recent studies that have investigated this concept. A South Australian non‐Indigenous PhD student and their research team sought to co‐design a Participatory Action Research Dadirri‐Ganma methodology, describing this co‐design process as an outcome in their paper. The authors concluded that this unique approach reflected mutual trustworthy partnerships based on collaboration over direction [28]. Similarly, these findings are consistent with researchers who worked with women from the Ngaanyatjarra Pitjantjatjara Yankunytjatjara region in remote central Australia to understand how to better service delivery for those experiencing sexual violence [41]. They found that a participatory action process that was flexible and adaptable to working with the needs of the community was essential when attempting to understand issues that the community may experience. These studies have demonstrated that following a methodology based on culturally safe principles over a fixed protocol created space for collaboration, mutual respect and knowledge sharing. In practice, this can mean multiple meetings between the non‐Indigenous researchers and Aboriginal people before sufficient understanding can be built to develop authentic, genuine partnerships necessary for a successful co‐design process [28].

## 5 Conclusion

In summary, arriving at a culturally safe outcome can only be derived from implementing a culturally safe process. Further, this process can itself be the outcome. While yet to be measured, it would be interesting to assess the impact of implementing a culturally safe process on the participants that chose to share their stories and whether this had any psychosocial benefit. This would suggest two outcomes can arise from adopting a culturally safe process: one, the cultural adaptation of an intervention, and two, a decrease in subjective psychosocial distress post‐engagement in the process.

Non‐Indigenous researchers partnering with Aboriginal and Torres Strait Islander young people experiencing self‐harm and/or suicidal behaviours should remain ever cognisant of the impact of their role as part of the co‐design process. The way we implement cultural safety (e.g., respond to their disclosure of past trauma) can impact the very population we ultimately seek to support. A culturally safe process breeds a culturally safe outcome.

## Author Contributions

**Craig D'Mello:** conceptualization (lead), writing – original draft (lead), methodology, investigation, formal analysis, writing – review and editing. **Helen Milroy:** conceptualization (supporting), methodology, writing – review and editing. **Alana Papageorgiou:** methodology, writing – review and editing. **Mathew Coleman:** writing – review and editing. **Patricia Dudgeon:** writing – review and editing. **Paulette Anderson:** investigation, writing – review and editing. **David Batty:** investigation, writing – review and editing. **Ashleigh Lin:** conceptualization (supporting), methodology, writing – review and editing.

## Funding

Craig D'Mello is funded by Suicide Prevention Australia. This project was supported by the University of Western Australia's Young Lives Matter Foundation. Professor Ashleigh Lin is funded by an NHMRC Emerging Leadership Fellowship (#2010063). No external or internal funding/supportive body was involved in the process of this manuscript.

## Ethics Statement

This project received ethics approval from the Western Australian Aboriginal Health Ethics Committee (HREC 1142).

## Conflicts of Interest

The authors declare no conflicts of interest.

## Data Availability

The authors have nothing to report.
